# Kappa-Opioid Receptor Antagonism Prolongs the Antidepressant Effects of Ketamine in Adult Mice with Depression-like Behavior Induced by Adolescent Chronic Unpredictable Stress

**DOI:** 10.3390/ijms27062815

**Published:** 2026-03-20

**Authors:** Ana Zivanovic, Milos Mitic, Iva Lukic, Emilija Glavonic, Miroslav Adzic, Sanja Ivkovic

**Affiliations:** Vinca–Institute for Nuclear Sciences, National Institute of Republic of Serbia, University of Belgrade, Mike Petrovica Alasa 12-14, 11000 Belgrade, Serbia; anazivanovicmb@gmail.com (A.Z.); milos.mitic@vin.bg.ac.rs (M.M.); iva.lukic@vin.bg.ac.rs (I.L.); emilija.glavonic@vin.bg.ac.rs (E.G.); miraz@vin.bg.ac.rs (M.A.)

**Keywords:** chronic unpredictable stress (CUS), ketamine, κ-opioid receptor (KOR), norbinaltorphimine (nBNI), depression

## Abstract

Major depressive disorder (MDD) is a highly prevalent psychiatric illness for which rapid-acting antidepressants such as ketamine provide only transient benefit. Because κ-opioid receptor (KOR) signaling contributes to stress-related dysphoria and impaired neuroplasticity, we examined whether KOR antagonism could prolong ketamine’s antidepressant-like effects in a mouse model of adolescent chronic unpredictable stress (CUS). Male *C57BL/6J* mice (n = 10 per group for behavioral analyses) were exposed to CUS during adolescence and developed persistent depression-like behavior in adulthood. Mice with depressive-like behavior received a single injection of ketamine, the selective KOR antagonist norbinaltorphimine (nBNI), or their combination. Behavioral testing showed that all treatments reduced immobility in the tail suspension test (TST) 24 h post-administration; however, only the combined ketamine/nBNI treatment maintained antidepressant-like effects one week post-treatment. Molecular analyses (n = 4–8 per group) were conducted at this single time point, one week post-treatment, to characterize region-specific signaling states in the prefrontal cortex, hippocampus, and striatum, focusing on ERK, AKT, JNK, mTOR, and BDNF pathways. These molecular findings represent correlates of sustained behavioral effects rather than evidence of causal mechanisms. Together, the data indicate that concurrent KOR antagonism is associated with prolonged antidepressant response to ketamine in stress-exposed male mice and with distinct region-dependent signaling profiles at one week post-treatment. Further studies are needed to establish mechanistic causality and confirm the possible applicability of these findings.

## 1. Introduction

Major depressive disorder (MDD) affects more than 300 million people worldwide and remains a leading cause of disability [1]. Although several pharmacological treatments are available, many patients fail to achieve sustained remission, highlighting the need for faster-acting and longer-lasting therapeutic strategies [2,3]. Adolescence represents a critical developmental window during which exposure to chronic stress can produce long-lasting alterations in brain circuits regulating mood, reward, and cognition, thereby increasing vulnerability to depression in adulthood [4,5,6,7].

Ketamine, a noncompetitive N-methyl-D-aspartate (NMDA) receptor antagonist, has emerged as a rapid-acting antidepressant capable of producing symptom relief within hours, even in treatment-resistant patients [8,9]. At subanesthetic doses, ketamine preferentially blocks NMDA receptors on inhibitory GABA interneurons [10], resulting in disinhibition of glutamatergic pyramidal neurons. This leads to a transient glutamate surge at the postsynaptic site, activating α-amino-3-hydroxy-5-methyl-4-isoxazolepropionic acid (AMPA) receptors and subsequent brain-derived neurotrophic factor (BDNF)–mammalian target of rapamycin (mTOR) signaling [11,12]. BDNF activation further stimulates mTOR through extracellular signal-regulated kinase (ERK)- and protein kinase B (AKT)-dependent mechanisms, forming a positive feedback loop that promotes synaptogenesis and enhances neuronal connectivity [13,14]. However, the antidepressant effects of ketamine are often short-lived, typically waning within days to a week after a single administration [15,16]. Strategies that extend the durability of ketamine’s therapeutic actions, therefore, represent an important area of investigation.

One system strongly implicated in stress-induced negative affect is the dynorphin/κ-opioid receptor (KOR) system. Activation of KOR signaling has been linked to dysphoria, anhedonia, and stress-related behavioral adaptations in both rodents and humans [17,18,19,20]. While kappa-opioid receptor (KOR) activation produces dysphoria and anhedonia, KOR antagonism exerts antidepressant-like and anti-stress effects [21]. Although the involvement of opioid signaling in stress responsivity and ketamine’s antidepressant actions has been previously documented [19,22], it remains unclear whether sustained KOR blockade can extend the temporal persistence of ketamine’s behavioral effects under conditions of prior chronic stress exposure. Among selective KOR antagonists, norbinaltorphimine (nBNI) exhibits strong specificity for KOR, with over 100-fold greater selectivity than for the mu-opioid receptor (MOR) [23,24]. It is important to note that although nBNI is not a clinically approved therapeutic agent, it has complex pharmacodynamic properties and a prolonged duration of action [25,26], which make it particularly suitable as a preclinical tool compound for probing the functional consequences of sustained KOR blockade. The present study seeks to test a specific durability-oriented framework: whether administration of a long-acting KOR antagonist, nBNI, can prolong the antidepressant-like effects of a single ketamine dose in mice previously exposed to adolescent chronic unpredictable stress (CUS), with behavioral outcomes evaluated up to one week post-treatment. This approach is intended to address a mechanistic durability question rather than to propose nBNI itself as a translational candidate.

We hypothesized that, compared with ketamine alone, combined administration of ketamine and the long-acting KOR antagonist norbinaltorphimine (nBNI) would be associated with (a) longer persistence of antidepressant-like behavioral effects and (b) region-specific changes in intracellular signaling pathways linked to stress responses and synaptic plasticity in the prefrontal cortex, hippocampus, and striatum. These findings may inform future investigations into whether clinically viable KOR-targeting strategies could influence the durability of rapid-acting antidepressant responses.

## 2. Results

### 2.1. Kappa Opioid Receptor Inhibition Prolongs the Antidepressant Effects of Ketamine in Mice Exposed to Chronic Unpredictable Stress

**Figure 1:**
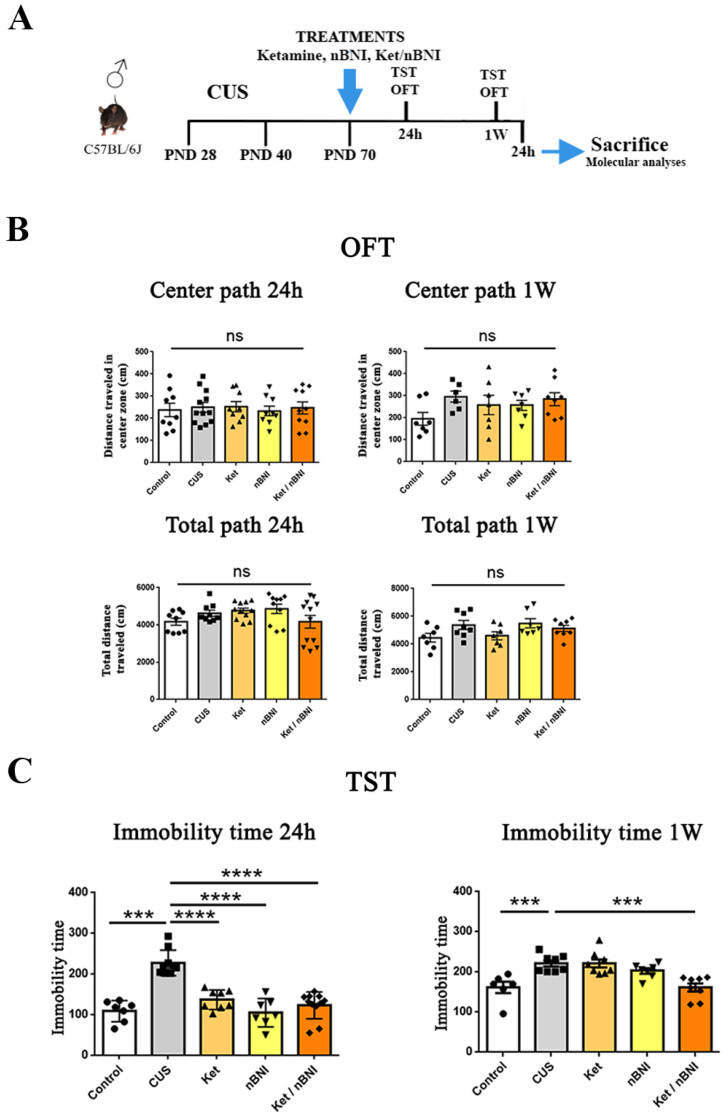
Experimental design and behavioral tests (OFT and TST) performance following CUS and treatments. (**A**) Time schedule/graphical representation of the experimental paradigm and behavioral tests (OFT and TST at 24 h and 1 w + 24 h after treatments), sacrifice, and molecular analyses (24 h after the last TST). Abbreviations—CUS: chronic unpredictable stress; PND 28: postnatal day 28; PND 40: postnatal day 40; PND 70: postnatal day 70. (**B**) Distance traveled in the center zone (central path) and total distance traveled (total path) in the OFT were measured 24 h and one week after treatments. (**C**) Immobility time in the TST was assessed 24 h and one week after treatment. Differences among all groups were analyzed by one-way ANOVA, with post hoc comparisons made against the CUS group. n = 10. Data are expressed as mean ± SEM. *** *p* < 0.001, **** *p* < 0.0001. Abbreviations: OFT, open field test; TST, tail suspension test; CUS, chronic unpredictable stress; Ket, ketamine; nBNI, norbinaltorphimine; Ket/nBNI, combination treatment with ketamine and norbinaltorphimine; ns, not significant.

The impact of chronic unpredictable stress (CUS) and pharmacological treatments on locomotor activity and depression-like behaviors was assessed using established behavioral paradigms: the open field test (OFT) [27] and the tail suspension test (TST) [28]. No significant differences were detected in the OFT parameters—total path length and center path—between the control and CUS groups or among the treatment groups at either 24 h or one week post-treatment (Figure 1B).

Depressive-like behavior was characterized by the TST at 24 h and one week post-treatment. TST was selected as the main readout because it is a widely validated, highly sensitive paradigm for detecting the effects of rapid-acting antidepressants like ketamine in C57BL/6J mice [28,29]. Given the large number of animals and the requirements of the experimental design, TST was selected as the most appropriate, as it allows rapid and reliable assessment of behavioral despair and is well-suited for high-throughput experimental design [28,30]. CUS exposure significantly increased immobility time in stressed animals at both 24 h (mean difference 98.3; Cohen’s d = 3.13; 95% CI 48.40 to 137.9) and one week (mean difference 59.75; Cohen’s d = 2.24; 95% CI 22.97 to 96.53) compared to controls (Figure 1C).

To evaluate the effects of CUS and pharmacological interventions—including individual (ketamine or nBNI) and combined (ketamine/nBNI) treatments—on locomotor activity and depression-like behaviors, behavioral tests (BTs) were conducted 24 h and one week post-treatment time points using the open field test (OFT) and the tail suspension test (TST). The experimental timeline is illustrated in Figure 1A.

Twenty-four hours post-treatment, all interventions significantly reduced immobility compared with the CUS group (ketamine: mean difference = −68.92; Cohen’s d = 2.22; 95% CI 27.21 to 110.6; nBNI: mean difference = −94.38; Cohen’s d = 2.21; 95% CI 52.67 to 110.6; Ket/nBNI: mean difference = −83.64; Cohen’s d = 1.91; 95% CI 44.46 to 122.8), confirming their short-term antidepressant efficacy (Figure 1C, left). However, at one week post-treatment, neither individual treatment maintained the antidepressant effect (Figure 1B). In contrast, the combined Ket/nBNI treatment produced a sustained reduction in immobility, effectively restoring it to control levels (Ket/nBNI: mean difference = −60.25; Cohen’s d = 2.63; 95% CI 26.20 to 94.30), demonstrating a prolonged antidepressant effect (Figure 1C, right).

### 2.2. Molecular Effects Associated with Chronic Unpredictable Stress and Drug Treatments

To delineate the molecular substrates associated with the behavioral effects of chronic stress and pharmacological intervention, we examined key intracellular signaling pathways governing neuroplasticity and stress adaptation within the prefrontal cortex (PFC), hippocampus (HIPP), and striatum (STR). Western blot analyses were employed to quantify the activation state of ERK, AKT, JNK, and mTOR kinases, conveyed as phosphorylated-to-total protein ratios. Together with BDNF signaling, these proteins collectively regulate synaptic plasticity, neuronal resilience, and affective homeostasis [31,32].

#### 2.2.1. Prefrontal Cortex

**Figure 2:**
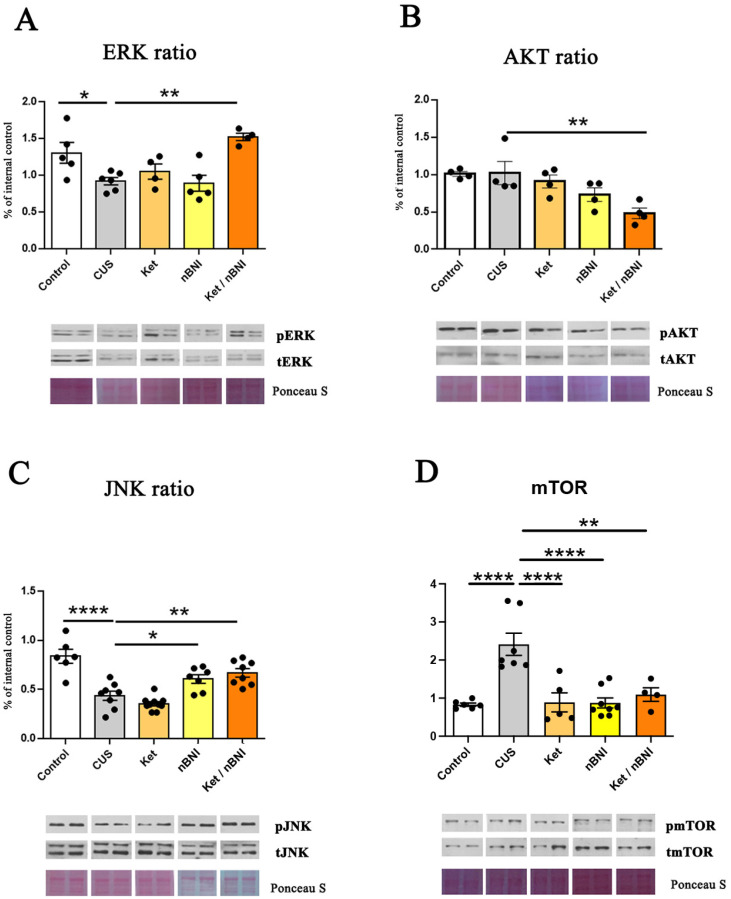
Effects of CUS and treatments on ERK, AKT, JNK, and mTOR activation in the PFC. (**A**) Phosphorylation ratio of ERK (p-ERK/ERK). (**B**) Phosphorylation ratio of AKT (p-AKT/AKT). (**C**) Phosphorylation ratio of JNK (p-JNK/JNK). (**D**) Phosphorylation ratio of mTOR (p-mTOR/mTOR). Differences among all groups were analyzed by one-way ANOVA, with Tukey post hoc comparisons made against the CUS group. n = 4–10. Data are expressed as mean ± SEM. * *p* < 0.05, ** *p* < 0.01, **** *p* < 0.0001. Abbreviations: CUS, chronic unpredictable stress; Ket, ketamine; nBNI, norbinaltorphimine; Ket/nBNI, combination treatment with ketamine and norbinaltorphimine.

CUS markedly reduced ERK activation (mean difference = −0.38, 95% CI [0.73, 0.04], Cohen’s d = 1.96) in the PFC, while AKT signaling was not affected. Among treatments, only the combined Ket/nBNI treatment restored ERK activity to control levels (mean difference = −0.60; 95% CI [−0.97, −0.23], Cohen’s d = 6.1), while single treatments had no effect (Figure 2A,B). Similarly, only Ket/nBNI treatment affected AKT signaling and reduced its activation (mean difference = 0.54, 95% CI [0.17, 0.90], Cohen’s d = 3.5). These findings suggest that simultaneous NMDAR and KOR inhibition exerts the normalization of ERK activity while reducing AKT activity (Figure 2A,B). CUS exposure significantly decreased JNK activation compared to controls (mean difference = −0.40, 95% CI [−0.57, −0.23], Cohen’s d = 2.67) (Figure 2C). While nBNI and Ket/nBNI treatments significantly increased JNK activation compared to CUS (nBNI: mean difference = −0.17, 95% CI [−0.33, −0.007], Cohen’s d = 1.48; Ket/nBNI: mean difference = −0.23, 95% CI [−0.39, −0.08], Cohen’s d = 2.2), ketamine had no effect. The mTOR activity was elevated in the PFC in CUS mice (mean difference 1.584; 95% CI [0.86, 2.31], Cohen’s d = 4.5) and was normalized similarly by all the treatments (Ket: mean difference = 1.52, 95% CI [0.76, 0.2.3], Cohen’s d = 2.48; nBNI: mean difference = 1.538, 95% CI [0.86, 2.21], Cohen’s d = 2.51; Ket/nBNI: mean difference = 1.32, 95% CI [0.49, 2.14], Cohen’s d = 6.55) (Figure 2D).

#### 2.2.2. Hippocampus

**Figure 3:**
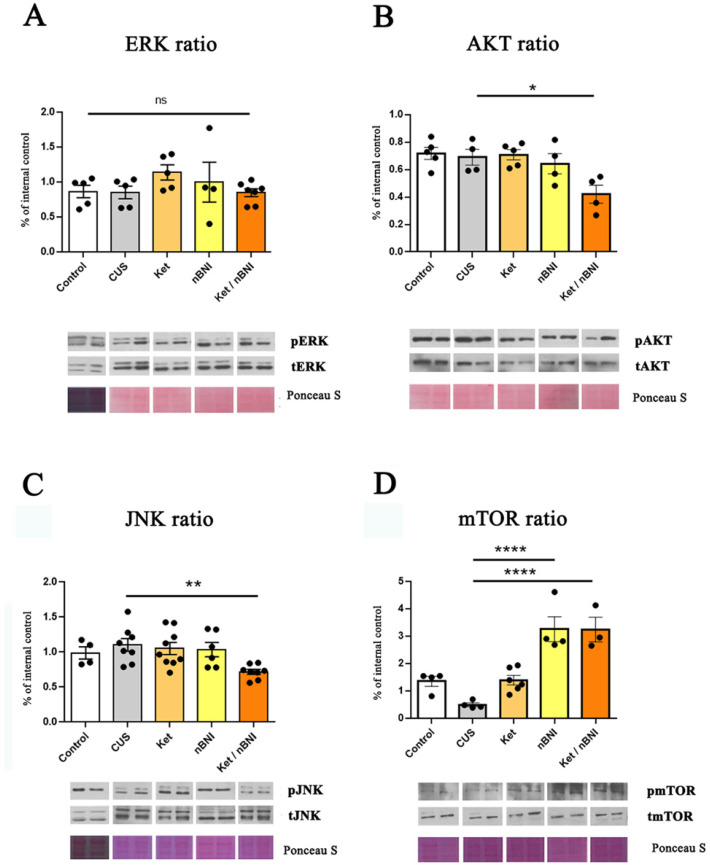
Effects of CUS and treatments on ERK, AKT, JNK, and mTOR activation in the hippocampus. (**A**) Phosphorylation ratio of ERK (p-ERK/ERK). (**B**) Phosphorylation ratio of AKT (p-AKT/AKT). (**C**) Phosphorylation ratio of JNK (p-JNK/JNK). (**D**) Phosphorylation ratio of mTOR (p-mTOR/mTOR). n = 4–10. Differences among all groups were analyzed by one-way ANOVA, with post hoc comparisons made against the CUS group. Data are expressed as mean ± SEM. * *p* < 0.05, ** *p* < 0.01, **** *p* < 0.0001, ns, not significant.

Neither ERK nor AKT signaling in the hippocampus was affected either with CUS or with any of the treatments except for the significant decrease in AKT following the Ket/nBNI treatment (mean difference = 0.27, 95% CI [0.053, 0.49], Cohen’s d = 2.8) (Figure 3A,B). Similarly, JNK and mTOR activation did not differ between control and CUS groups (Figure 3C,D). However, Ket/nBNI treatment significantly reduced JNK activation (mean difference = 0.48, 95% CI [0.19, 0.78], Cohen’s d = 5), while ketamine or nBNI alone had no effect (Figure 3C). In addition, the combined Ket/nBNI treatment robustly increased mTOR activation compared to CUS (mean difference = −2.66, 95% CI [−3.75, −1.56], Cohen’s d = 6.55), as did the treatment with nBNI (mean difference = −2.67, 95% CI [−3.67, −1.67], Cohen’s d = 4.58). At the same time, ketamine treatment alone did not significantly affect mTOR signaling in the hippocampus (Figure 3D).

#### 2.2.3. Striatum

**Figure 4:**
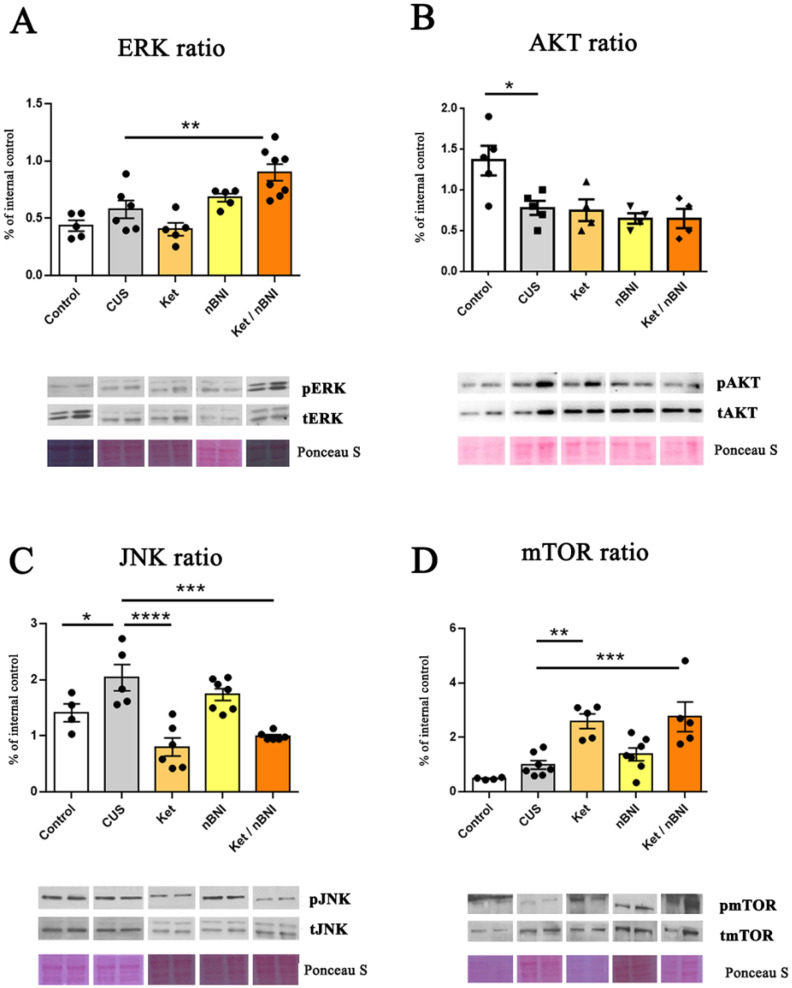
Effects of CUS and treatments on ERK, AKT, JNK, and mTOR activation in the striatum. (**A**) Phosphorylation ratio of ERK (p-ERK/ERK). (**B**) Phosphorylation ratio of AKT (p-AKT/AKT). (**C**) Phosphorylation ratio of JNK (p-JNK/JNK). (**D**) Phosphorylation ratio of mTOR (p-mTOR/mTOR). n = 4–10. Differences among all groups were analyzed by one-way ANOVA, with post hoc comparisons made against the CUS group. Data are expressed as mean ± SEM. * *p* < 0.05, ** *p* < 0.01, *** *p* < 0.001, **** *p* < 0.0001.

While ERK activation in the striatum was unaffected by CUS, the AKT activation was significantly reduced (mean difference = −0.58, 95% CI [−1.04, −0.12], Cohen’s d = 2.32) (Figure 4A,B). Only the combined Ket/nBNI treatment was able to increase ERK activation compared to CUS (mean difference = −0.32, 95% CI [−0.55, −0.09], Cohen’s d = 1.78), while individual treatments did not have any effect. At the same time, none of the treatments restored AKT activity levels (Figure 4B). CUS exposure significantly increased JNK activation in the striatum (mean difference = 0.63, 95% CI [0.3, 1.22], Cohen’s d = 1.71), but did not affect mTOR activation (Figure 4C,D). The combined Ket/nBNI treatment significantly reduced JNK activation (mean difference = 1.04, 95% CI [0.50, 1.58], Cohen’s d = 4.04) and increased mTOR activity (mean difference = 0.50, 95% CI [−0.62, 1.63], Cohen’s d = 2), Interestingly, ketamine treatment alone produced a similar effect—a significant reduction in JNK activity (mean difference = 1.24, 95% CI [0.70, 1.78], Cohen’s d = 2.87) and concurrent increase in mTOR activity (mean difference = −1.60, 95% CI [−2.66, −0.55], Cohen’s d = 3.44). The treatment with nBNI alone had no effect on either JNK or mTOR signaling when compared to CUS (Figure 4C,D).

### 2.3. Correlations Between Molecular Markers

**Figure 5:**
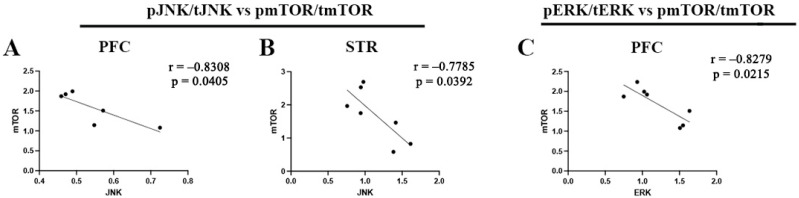
Pearson correlations between molecular markers following Ket/nBNI treatment. A significant negative correlation was observed between levels of JNK and mTOR activity in the PFC (**A**) and STR (**B**) and between levels of ERK and mTOR activity in the PFC (**C**) in animals receiving the combined Ket/nBNI treatment.

To further characterize signaling relationships associated with the combined Ket/nBNI treatment, correlation analyses were performed between upstream kinases and mTOR activation across brain regions (Figure 5). In both PFC and STR, JNK activation was inversely correlated with mTOR activation (r = −0.8308 and r = −0.7785, respectively) (Figure 5A,B), indicating that lower JNK activity was associated with higher mTOR activation in these regions. A similar inverse association was observed in the PFC between ERK and mTOR activation (r = −0.8279) (Figure 5C). Although these correlations do not establish direct cause-and-effect interactions, they suggest that the activity of these pathways changes in parallel under the combined treatment condition. Notably, the consistent negative relationship between JNK and mTOR across cortical and striatal regions represents a recurring molecular pattern linked with the treatment-associated signaling profile.

### 2.4. Regional Differences in Stress- and Treatment-Associated Modulation of BDNF

**Figure 6:**
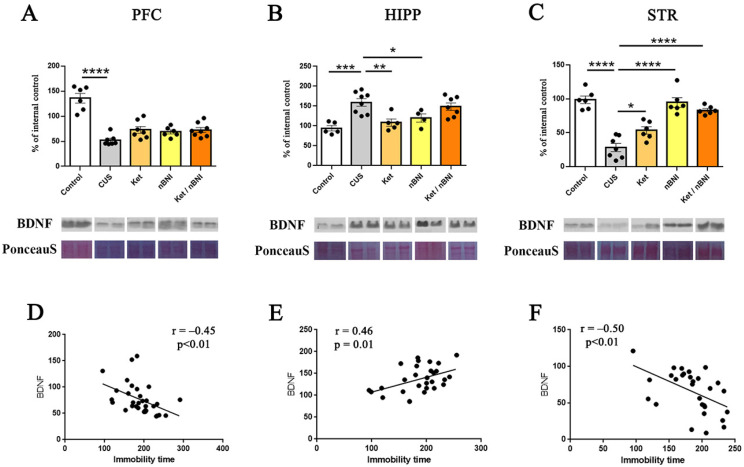
Effects of CUS and treatments on BDNF expression in PFC, HIPP, and STR. BDNF expression in the PFC (**A**), hippocampus (**B**), and striatum (**C**). Pearson correlations between BDNF expression and immobility time in the PFC (**D**), HIPP (**E**), and STR (**F**). n = 4–10. Differences among all groups were analyzed by one-way ANOVA, with post hoc comparisons made against the CUS group. Data are expressed as mean ± SEM. * *p* < 0.05, ** *p* < 0.01, *** *p* < 0.001, **** *p* < 0.0001.

In the PFC, CUS significantly reduced BDNF expression compared to the control group (mean difference = −83.75, 95% CI [−106.6, −60.88], Cohen’s d = 8.3) (Figure 6A). None of the treatments reversed the stress-induced alterations, indicating persistent impairment of neurotrophic signaling. In contrast, in the hippocampus, CUS significantly elevated BDNF expression relative to controls (mean difference = 65.02, 95% CI [30.41, 99.64], Cohen’s d = 8.3) (Figure 6B), suggesting a possible compensatory response to chronic stress. Surprisingly, the combined Ket/nBNI treatment did not have any effect on CUS-induced BDNF increase in the hippocampus, although ketamine and nBNI treatments were able to attenuate this increase (ketamine: mean difference = 50.72, 95% CI [16.11, 85.34], Cohen’s d = 2.34; nBNI: mean difference = 39.23, 95% CI [2.05, 76.4], Cohen’s d = 1.79). In the striatum, CUS profoundly suppressed BDNF expression (mean difference = −70.33, 95% CI [−90.62, −50.03], Cohen’s d = 5.3) (Figure 6C). Although all treatments significantly increased BDNF levels, the highest increase was achieved with Ket/nBNI and nBNI treatments (nBNI: mean difference = −66.45, 95% CI [−86.74, 46.15], Cohen’s d = 4.55; Ket/nBNI: mean difference = −55.01, 95% CI [−75.30, −34.71, Cohen’s d = 5.5) that fully counteracted the stress-induced BDNF suppression. Ketamine treatment only partially reversed this suppression, increasing BDNF levels (mean difference = −25.27, 95% CI [−45.56, 4.97], Cohen’s d = 1.91) (Figure 6C).

In order to explore the relationship between BDNF expression and behavioral performance across the examined brain structures (PFC, HIPP, and STR), correlation analyses were performed (Figure 6D–F). BDNF expression showed significant correlations with immobility time across all three brain structures, although the direction of these correlations differed between regions. In the PFC and STR, a significant negative correlation was observed (r = −0.45; r = −0.50, respectively) (Figure 6D,F), whereas in the hippocampus, the correlation was positive (r = 0.46) (Figure 6E).

## 3. Discussion

The present study examined whether κ-opioid receptor (KOR) antagonism can prolong the antidepressant-like effects of ketamine in adult male mice that developed depressive-like behavior following adolescent chronic unpredictable stress (CUS). While a single administration of ketamine, nBNI, or their combination produced short-term antidepressant-like effects 24 h after treatment, only the combined Ket/nBNI intervention maintained behavioral improvement one week later, when the effects of individual treatments had subsided. These findings support the hypothesis that inhibition of the dynorphin–KOR system enhances the durability of ketamine’s antidepressant-like actions in the context of prior adolescent stress exposure.

### 3.1. Long-Term Impact of Adolescent Stress on Adult Brain Signaling

The long-term consequences of adolescent CUS were evident in adulthood as persistent depressive-like behavior accompanied by robust, region-specific alterations in stress-related intracellular signaling pathways (Table 1). In the prefrontal cortex (PFC), adolescent stress was associated with reduced trophic support and disrupted kinase balance, consistent with impaired top-down regulation of affective and stress responses. ERK activation was reduced, consistent with chronic stress impairing trophic and survival signaling [33], whereas AKT signaling remained unchanged. Paradoxically, increased mTOR activation, despite reduced upstream kinase activity, may reflect long-term maladaptive circuit remodeling following stress [34]. Alternatively, it could be linked to stress-induced metabolic dysregulation and increased body weight, as chronic mTOR hyperactivation is known to promote anabolic processes and contribute to obesity [35]. JNK activity was markedly suppressed in the PFC, suggesting long-term downregulation of stress-responsive kinase activity following chronic stress, as was reported previously [36,37]. The long-term reduction in JNK activity may be associated with a rebound increase in mTOR signaling, but this shift occurred without restoration of upstream trophic support. Consistent with this, BDNF levels in the PFC remained significantly decreased long after CUS and showed a negative correlation with immobility time, suggesting that persistent deficits in neurotrophic signaling may contribute to the maintenance of depressive-like behavior [38].

In contrast to other regions, the hippocampus displayed a molecular profile consistent with long-term compensatory or metaplastic adaptation rather than a persistent acute stress state. Although chronic stress often reduces hippocampal BDNF [39,40,41], its direction and magnitude can vary with stress timing and duration, sometimes resulting in recovery or even upregulation [42]. In our study, elevated BDNF alongside preserved baseline mTOR activity suggests a region-specific adaptation emerging during prolonged recovery from adolescent stress.

Meanwhile, the striatum showed alterations in stress- and trophic-related signaling compatible with modified reward-circuit regulation. The striatum displayed a distinct stress-response pattern characterized by elevated JNK activation and reduced AKT signaling, with ERK and mTOR levels unchanged. This pattern may reflect altered stress sensitivity or signaling imbalance, potentially biasing the region toward pro-apoptotic or maladaptive signaling [43]. Finally, BDNF expression in the adult striatum was significantly reduced long-term following chronic stress, as reported previously [44,45].

The present findings of opposite-direction BDNF–behavior correlations across brain regions are consistent with a growing body of evidence indicating that stress-induced BDNF modulation is highly region-dependent and can participate in distinct adaptive or maladaptive responses depending on local circuit demands and stress history [46]. Mechanistically, BDNF levels are also highly dynamic and influenced by interactions with glucocorticoid signaling and other modulatory systems. Reviews of stress-related neuroplasticity illustrate that BDNF expression not only varies across brain structures but also fluctuates over the course of acute and chronic stress exposure and that these variations emerge in parallel with changes in glucocorticoid receptor signaling and downstream intracellular cascades that shape synaptic remodeling [47]. Under this framework, elevated hippocampal BDNF in stressed animals may reflect a compensatory or metaplastic process, especially following prolonged stress or recovery periods, while suppressed BDNF in prefrontal and striatal regions may index persistent trophic deficits related to stress pathology. Such region-specific BDNF dynamics are consistent with the notion that stress does not uniformly dampen or elevate neurotrophic signaling but instead reprograms plasticity responses according to the unique functional and receptor profiles of each circuit, thereby shaping both behavioral vulnerability and resilience [42].

Together, these findings support the idea that early-life stress leaves a long-lasting heterogeneous molecular imprint across corticolimbic and striatal networks, which may shape both vulnerability to depression and responsiveness to later interventions.

### 3.2. Region-Specific Molecular Changes Following the Combined Ket/nBNI Treatment

The sustained antidepressant-like effect observed after combined Ket/nBNI treatment was accompanied by region-specific modulation of intracellular pathways involved in stress adaptation and synaptic plasticity, including ERK, AKT, JNK, mTOR, and BDNF. The concurrent NMDA receptor and KOR blockade was associated with structure-dependent kinase changes converging on mTOR and BDNF regulation, rather than with a uniform molecular response.

Among the pathways examined, JNK—a stress-responsive kinase and downstream component of KOR-related signaling—was of particular interest. Given that stress has been reported to regulate JNK in a region-dependent manner [48,49], concurrent modulation of NMDA receptors and KOR may be associated with differential patterns of JNK-related signaling across corticolimbic and striatal circuits. In addition, prior work indicates that KOR blockade can be accompanied by alterations in components of the NMDA–BDNF–mTOR signaling cascade under stress conditions [50], suggesting that combined glutamatergic and KOR modulation may coincide with molecular states consistent with plasticity-related processes.

We deliberately selected a single 6 mg/kg dose of ketamine [51] for the following reasons: to balance antidepressant-like efficacy with minimization of nonspecific behavioral effects (e.g., hyperlocomotion or psychotomimetic-like activity) that can occur at higher or repeated doses [52]. Limiting the study to a single administration and defined post-treatment assessments reduced confounds related to repeated injections, cumulative drug exposure, and handling stress, thereby allowing clearer evaluation of the pharmacological interaction between ketamine and KOR antagonism. The one-week post-treatment time point was selected to assess behavioral persistence beyond acute pharmacological effects, as ketamine and its metabolites are cleared well before this interval [53,54]. Although nBNI has prolonged receptor-level activity [25], nBNI alone did not produce sustained behavioral effects at one week [55], supporting the interpretation that the combined treatment reflects longer-lasting functional adaptations rather than residual drug presence. Importantly, nBNI is not solely a neutral receptor antagonist but has been described as a biased ligand capable of recruiting JNK and producing prolonged functional desensitization of KOR [56,57]. Such prolonged receptor modulation has been associated with changes in neurotrophic signaling pathways, including mTOR and BDNF [29,58]. In the present context, these signaling alterations are interpreted as molecular correlates observed alongside sustained behavioral effects rather than as evidence of direct mechanistic mediation.

#### 3.2.1. PFC

In the PFC, the combined Ket/nBNI treatment was associated with alterations in signaling pathways upstream of mTOR (Table 2). ERK and JNK, but not AKT, showed changes in directions opposite to those observed under CUS exposure. These patterns corresponded to differences in mTOR-related signaling relative to stress conditions. Rather than reflecting uniform elevation of mTOR activity, the data indicate a redistribution of upstream kinase phosphorylation profiles in stress-exposed animals receiving the combined treatment. The partial overlap between the effects observed with ketamine and nBNI alone suggests that both glutamatergic and KOR-related signaling systems are represented in the observed molecular profiles. Persistent reductions in BDNF levels, despite changes in kinase phosphorylation, indicate that trophic markers and kinase signaling do not shift in parallel at this single time point and may reflect region-specific or temporally distinct adaptations.

#### 3.2.2. Hippocampus

The hippocampal signaling profile associated with the combined treatment differed from that observed in the PFC (Table 2). In this region, Ket/nBNI was associated with reduced AKT and JNK phosphorylation alongside increased mTOR phosphorylation. This pattern reflects a divergence between upstream kinase phosphorylation states and mTOR-related signaling at this single time point. Although feedback interactions downstream of mTORC1 have been described in the literature [59,60,61], the present data do not permit determination of pathway directionality. Given that CUS exposure was associated with elevated hippocampal BDNF levels, the additional changes in mTOR phosphorylation observed following treatment represent a distinct molecular profile relative to stress alone, without establishing whether these shifts reflect restoration, compensation, or alternative adaptations.

#### 3.2.3. Striatum

In the striatum, the combined treatment was associated with a molecular pattern distinct from both cortical and hippocampal profiles (Table 2). CUS exposure increased JNK phosphorylation in this region, and Ket/nBNI-treated animals showed lower JNK phosphorylation relative to CUS, accompanied by higher mTOR phosphorylation and BDNF levels at the one-week time point. These concurrent changes indicate a region-specific signaling configuration within striatal tissue. While the striatum is implicated in motivation and reward-related processes, the present data do not establish functional consequences of the observed molecular differences. The treatment with ketamine alone showed a phosphorylation pattern partially overlapping with that of the combined treatment, whereas nBNI alone produced comparatively limited changes in these markers. These findings describe treatment-associated molecular signatures but do not determine the mechanistic hierarchy or causal contribution of individual pathways.

### 3.3. Integration of Correlation Analyses

The correlation patterns observed after the combined Ket/nBNI treatment provide additional context for interpreting the region-specific signaling adaptations. In both the PFC and striatum, the inverse association between JNK and mTOR activation indicates that lower JNK activity coincided with greater mTOR pathway engagement under the treatment condition. Although correlational analyses do not establish regulatory directionality, the recurrence of this relationship across two anatomically and functionally distinct regions suggests a coordinated shift in signaling balance, whereby reduced stress-associated kinase activity accompanies enhanced plasticity-related pathway activation. A similar inverse relationship between ERK and mTOR in the PFC further demonstrates that upstream kinase activity does not uniformly scale with mTOR output, underscoring the complexity of intracellular signal integration within stress-responsive circuits.

Importantly, these associations should not be interpreted as evidence of direct inhibitory interactions between pathways but rather as coordinated signaling states associated with the combined pharmacological intervention. ERK and mTOR are both downstream effectors of glutamatergic and trophic signaling and can influence each other through intermediary regulators. In addition, strong mTORC1 activation is known to engage negative feedback mechanisms involving S6K1 and IRS proteins, which can reshape upstream AKT and MAPK signaling [61]. The inverse ERK–mTOR relationship observed in the PFC may therefore reflect network-level feedback and pathway redistribution rather than a linear regulatory interaction.

Collectively, these data propose a model in which Ket/nBNI treatment engages region-specific plasticity mechanisms. While the PFC and hippocampus show signs of normalized signaling, the striatum exhibits a unique profile of mTOR and BDNF upregulation that parallels the sustained antidepressant-like effects. While direct functional validation is needed, we posit that this distinct upregulation of striatal plasticity may contribute to the extended therapeutic duration observed in the combined treatment group.

Therefore, the persistence of behavioral improvements observed with the combined Ket/nBNI treatment cannot be attributed solely to ketamine or interpreted as strict pharmacological synergy. Rather, the findings should be interpreted as a combinatorial association in which sustained receptor-level modulation by nBNI may create a permissive molecular environment that supports the prolonged engagement of plasticity-related pathways by a single ketamine dose.

### 3.4. Limitations and Future Directions

Although our study provides insights into the complex molecular consequences of chronic unpredictable stress (CUS) and the therapeutic potential of ketamine and KOR antagonism, several limitations should be considered when interpreting the findings.

First, experiments were conducted exclusively in male mice, which limits conclusions regarding sex-dependent behavioral and molecular responses. However, it is important to emphasize that the results remain internally valid and biologically meaningful within this experimental context, as male rodents are widely used and well-characterized in preclinical stress and ketamine research. Substantial evidence indicates that females may exhibit distinct—and in some cases divergent—responses to ketamine and stress-related manipulations. For example, ketamine has been reported to produce sustained antidepressant-like effects in males while inducing delayed anxiety- or depressive-like behaviors in females [62]. Female rodents may also respond to lower ketamine doses, with ovarian hormones influencing treatment sensitivity across the estrous cycle [63,64]. Accordingly, inclusion of both sexes in future studies will be essential to determine the generalizability of the present findings rather than to establish their validity [6].

Second, the study employed a single ketamine dose and a single defined post-treatment time point. This design limits inference regarding repeated dosing regimens, dose–response relationships, and longer-term trajectories beyond one week. Clinically, ketamine is often administered repeatedly, and whether sustained KOR antagonism would extend durability under such paradigms remains to be determined. Nevertheless, isolating a single administration allowed us to specifically test the durability of an acute pharmacological interaction without confounds related to tolerance, cumulative neuroplastic adaptations, or repeated handling stress. This focused design strengthens interpretability regarding persistence beyond acute exposure.

Third, molecular analyses were performed at a single post-treatment time point, which limits conclusions regarding the temporal evolution of the observed signaling changes. Nevertheless, the selected time point was sufficient to capture stable behavioral effects and associated molecular signatures, providing a meaningful snapshot of stress- and treatment-related adaptations. Future studies incorporating longitudinal molecular profiling will help clarify the persistence and progression of these changes.

Fourth, the behavioral assessment in this study relied primarily on the tail suspension test (TST). While TST is a robust, widely validated, and highly sensitive paradigm for detecting the effects of rapid-acting antidepressants like ketamine in a large number of experimental animals, we acknowledge that it focuses on immobility as a measure of behavioral despair. The inclusion of additional behavioral paradigms, such as the sucrose preference test to assess anhedonia or the forced swim test, would provide a more comprehensive behavioral profile in future investigations. In addition, repeated behavioral testing may act as a stressor, potentially influencing molecular signaling [65]. To control for this, all groups—including controls—underwent the same testing schedule, ensuring that observed molecular differences reflect treatment and stress effects rather than unequal test exposure. Therefore, molecular findings should be interpreted as reflecting both treatment/stress and the impact of repeated testing.

Finally, although coordinated molecular changes were observed across multiple brain regions following combined ketamine and KOR antagonism, the present study was not designed to directly test mechanistic causality. Potential contributions of receptor-level interactions, intracellular signaling convergence, or circuit- and cell-type-specific processes therefore remain to be examined. Importantly, the region-specific signaling patterns identified here provide a focused and testable framework for future hypothesis-driven mechanistic studies.

Despite these limitations, the findings in this study provide novel preclinical evidence that KOR antagonism can prolong the antidepressant-like effects of ketamine and identify region-specific molecular adaptations associated with sustained behavioral improvement.

## 4. Materials and Methods

### 4.1. Animals

All experiments were conducted on adolescent male C57BL/6J mice acquired from the Jackson Laboratory and bred in our facilities. Animals were provided with commercial food pellets and water ad libitum and maintained on a 12 h light/dark cycle in a temperature-controlled environment (20 ± 2 °C). The animals were housed in groups of three to four per cage. All animal procedures were approved by the Ethical Committee of the VINCA Institute of Nuclear Sciences (323-07-03268/023-05), in accordance with the guidelines set by the European Communities Council Directive (2010/63/EU) for animal experiments.

During the experiment, adolescent animals from different litters were randomly divided into two main groups: a control group and a group exposed to chronic unpredictable stress (CUS). An investigator not involved in behavioral testing or molecular analyses randomly assigned animals to the control and CUS groups, making sure that animals in each group were from different litters and from different cages. A similar principle of randomization was applied for the allocation of CUS animals to treatment groups (as described in Section 4.3).

### 4.2. Chronic Unpredictable Stress (CUS)

Chronic stress is a major contributing factor in the development of mood disorders such as depression and anxiety, often leading to persistent behavioral and neurobiological alterations. To explore these effects, we employed the chronic unpredictable stress (CUS) paradigm, a well-established and widely validated model for inducing depression-like phenotypes in rodents [6,7,66].

Adolescent male mice were exposed to CUS for 12 consecutive days (Table 3), starting on postnatal day 28 (P28). The 12-day CUS protocol was selected based on prior validation in adolescent mice [6,7,66], a developmental period characterized by heightened stress sensitivity, during which shorter stress paradigms are sufficient to induce robust and persistent depressive-like behavioral phenotypes. Stressors were administered three times daily during the light phase (morning, afternoon, and evening) in designated procedure rooms. A variety of physical and social stressors were used each day to prevent habituation and ensure sustained stress responses. Between stressors, animals were returned to their home cages. Following the CUS procedure, mice were given a 30-day rest period (P40–P70) to allow maturation into adulthood before further testing and treatment.

### 4.3. Experimental Groups and Treatments

Animals not exposed to the CUS were used as controls (n = 10). To ensure consistency across all animals, the control group received an acute mass-adjusted volume of vehicle (0.9% NaCl) by intraperitoneal (i.p.) injection.

Drug administration: Animals exposed to the CUS were randomly divided into 4 groups (n = 10 per group) for the i.p. drug administration on P70: I CUS group was treated with the mass-adjusted volume of vehicle (saline, 0.9% NaCl); II Ket group was treated with ketamine (Ketamidor, Richter Pharma AG, Wels, Austria; dosage of 6 mg/kg) diluted in saline; III nBNI group was i.p. injected with nBNI (Sigma-Aldrich, St. Louis, MO, USA; dosage of 10 mg/kg) diluted in saline [56]; IV Ket/nBNI group was i.p. injected first with ketamine (6 mg/kg) and then with nBNI (10 mg/kg) diluted in saline, 30 min apart. A dose of 6 mg/kg ketamine was chosen based on previous studies demonstrating effective antidepressant-like effects in mice at this dose while maintaining a subanesthetic profile [51].

To evaluate the effects of CUS and pharmacological interventions—including individual (ketamine or nBNI) and combined (ketamine/nBNI) treatments—on locomotor activity and depression-like behaviors, behavioral tests (BTs) were conducted 24 h and one week post-treatment using the open field test (OFT) and the tail suspension test (TST). After the last BT was performed at one week post-treatment, the animals rested for 24 h and were then sacrificed, and brain structures (prefrontal cortex, hippocampus, and striatum) were isolated and immediately frozen at −80 °C. The experimental design is presented in Figure 1A. Molecular analyses (n = 8) were conducted 24 h after the last TST (which itself was conducted one week post-treatment) in order to minimize acute behavioral-test–induced signaling effects. In addition, all control animals used in the study underwent the same testing regimen.

### 4.4. Body Weight Measurement

Body weight (g) was measured at three time points: at the beginning of adolescence and immediately before the onset of the CUS protocol (P28), at the end of the 12-day CUS exposure (P40), and after the rest period when mice reached adulthood (P70). Monitoring of body weight served as an indicator of general health and as a physiological marker of chronic stress exposure. Measurements were performed individually using a precision digital scale at consistent times of day to minimize variability. Reduced body weight is a well-established physiological indicator of chronic stress in rodents [67].

**Figure 7:**
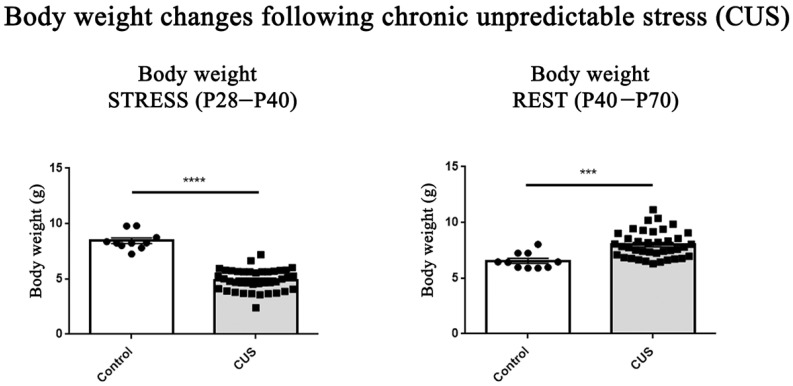
Body weight (g) changes in control and CUS-exposed adolescent mice. CUS mice showed significant weight loss at P40 (****, *p* < 0.0001) (**left**) and increased weight at P70 relative to controls (***, *p* < 0.001) (**right**). Data are mean ± SEM; unpaired Student *t*-test. Abbreviations—CUS: chronic unpredictable stress; P28: postnatal day 28; P40: postnatal day 40; P70: postnatal day 70.

CUS-exposed adolescent mice (P40) exhibited a significant reduction in body weight compared with control animals (*p* < 0.0001, Student *t*-test) (Figure 7), confirming the efficacy of the chronic stress procedure. However, by P70, CUS mice showed a marked increase in body weight relative to control animals (*p* < 0.001), indicating the greater weight gain during the rest period.

### 4.5. Behavioral Tests

#### 4.5.1. Open Field Test

The OFT was conducted in a white arena measuring 52 cm × 52 cm. Mice were placed in the arena and allowed 10 min to explore. A video tracking system recorded their movements, and the data was saved to a computer for subsequent analysis using TSE VideoMot 2 software (TSE Systems, Bad Homburg, Germany). The following parameters, as a measure of locomotor activity and anxiety-like behavior, were calculated: total distance traveled (total path) and time spent in the central area of the arena (center path). The open-field test was repeated twice: 24 h and 7 days after drug treatment. After each testing session, mice were returned to their home cages before proceeding to the TST [27].

#### 4.5.2. Tail Suspension Test

In the tail suspension test (TST), each mouse was suspended by its tail using adhesive tape, positioned so that it hung freely above the ground. To prevent visual or social interaction between animals, each mouse was placed in an individual compartment. The test lasted for 6 min, and the entire session was recorded using a video camera for subsequent behavioral scoring. The duration of immobility was quantified as an index of depressive-like behavior (behavioral despair or helplessness). Behavioral analysis was performed offline by two trained observers blinded to the experimental groups. After each session, the apparatus was thoroughly cleaned with a sterilizing solution to remove any olfactory cues [28].

### 4.6. Western Blot Analysis

The frozen brain tissues (n = 8) were then weighed and homogenized (1:10, *w*/*v*) in ice-cold 50 mM Tris-HCl (pH 7.5) buffer containing 150 mM NaCl, 1% NP-40, 0.1% SDS, 10 mM EDTA (pH 8), 10 mM EGTA (pH 7.2), 0.5% Triton x-100, and phosphatase inhibitors (20 mM b-glycerophosphate, 5 mM Na_4_P_2_O_7_ × 10 H_2_O, 2 mM Na_3_VO_4_, 25 mM NaF) with 30 strokes of a Potter Elvehjem Teflon-glass homogenizer. Each sample was centrifuged for 30 min at 14,000 rpm at 4 °C, in order to obtain the supernatant. The samples were subsequently frozen and stored at −80 °C prior to performing Western blot analysis.

Protein concentration was determined using the method of Markwell [68], and samples were incubated for 5 min at 100 °C in an appropriate amount of denaturing buffer, as described by Laemmli [69]. Forty μg of protein was subjected to sodium dodecyl sulfate-polyacrylamide gel electrophoresis (SDS-PAGE) using 7.5%, 10%, or 12% gels. The proteins were then transferred to a polyvinylidene fluoride (PVDF) membrane (Immobilon-P, Millipore, Darmstadt, Germany) using a Transblot transfer system (Bio-Rad, Hercules, CA, USA). Following transfer, the membrane was blocked with 5% non-fat milk in phosphate-buffered saline (PBS) for 1 h at room temperature. The membrane was then incubated overnight at 4 °C with primary antibodies diluted in 2.5% non-fat milk in PBS-Tween (PBS-T), as listed in Table 4. After overnight incubation with the primary antibodies, the membrane was washed three times with PBS-T. After incubation, membranes were washed three times with PBS-T and then incubated with HRP-conjugated secondary antibodies (rabbit anti-mouse IgG-HRP and goat anti-rabbit IgG-HRP; Abcam, Cambridge, UK). Immunoreactive bands were visualized using enhanced chemiluminescence (ECL) with SuperSignal Pico or Femto substrates (Thermo Scientific, Waltham, MA, USA) and exposed to X-ray film (Amersham Hyperfilm ECL, Cytiva, Wilmington, DE, USA).

Chemiluminescent signals from the immunoblots were detected and quantified using ImageJ analysis software (1.53k, NIH, Bethesda, MD, USA) for densitometry of protein bands on X-ray film. After quantification, protein levels were normalized to Ponceau S-stained total protein bands (40–70 kDa) as a loading control. To enable comparison across different gels, an internal reference standard (IRS)—an identical sample loaded on every gel—was used. For Western blot analyses, samples were coded prior to processing and quantification, and the investigator performing densitometric analyses was blinded to group identity. Relative protein expression in each sample was calculated as a percentage of IRS, ensuring consistency and allowing quantitative comparison between gels.

### 4.7. Statistical Analysis

Data were analyzed using the Prism program (GraphPad Prism, Software, v.6, La Jolla, CA, USA). Data distributions were assessed for normality (by the Shapiro–Wilk test) and for homogeneity of variance (by the Brown–Forsythe test) prior to analysis. Sample sizes for the study were determined using a priori power analysis based on our previous experimental evidence of CUS behavioral effects (power = 80% at α = 0.05). The comparison between the two experimental groups was performed by an unpaired Student’s *t*-test. For comparing multiple groups, one-way analyses of variance (ANOVA) were performed, followed by Dunnett’s post hoc test to determine statistical significance between the CUS group and other experimental groups. For molecular analyses, 95% confidence intervals for mean differences and effect sizes (Cohen’s d) were reported as indicators of the precision and magnitude of the observed effects. According to Cohen’s guidelines [70], Cohen’s d values greater than 0.8 were interpreted as indicators of a large effect. The outliers were excluded using the criteria median ± 2 MAD [71], and additional exclusions were made in cases of technical issues or sample loss. Pearson correlation coefficients (r) were calculated to assess the associations between expression levels of different proteins and between immobility time and protein expression levels. All data are expressed as mean ± standard error of the mean (SEM). The test was considered significant when *p* < 0.05.

## 5. Conclusions

In summary, this study demonstrates that sustained κ-opioid receptor blockade with the long-acting antagonist nBNI is associated with prolonged antidepressant-like effects of a single ketamine dose in a mouse model of adolescent chronic stress. The persistence of behavioral improvement one week after administration—beyond the established pharmacokinetic presence of ketamine—highlights a durability-oriented interaction between glutamatergic and KOR-related signaling systems. Region-specific molecular profiles identified in the prefrontal cortex, hippocampus, and striatum provide a defined snapshot of intracellular signaling states accompanying sustained behavioral effects; however, these molecular changes should be interpreted as correlates rather than mechanistic drivers.

Although the male-only cohort, single-dose design, limited behavioral battery, and single molecular time point constrain generalizability and translational extrapolation, this focused framework allowed a controlled examination of durability in a stress-sensitized phenotype. As such, the findings offer preclinical proof of concept that sustained modulation of the dynorphin–KOR system may influence the temporal persistence of rapid-acting antidepressant responses. Overall, the study provides encouraging support for combinatorial strategies targeting glutamatergic and opioid systems as a means of enhancing antidepressant durability, while underscoring the need for broader behavioral validation, sex-inclusive designs, and mechanistic expansion in future investigations.

## Figures and Tables

**Table 1:** Summary of regional molecular changes under CUS.

CUS	PFC	HIPP	STR
ERK	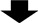	ns	ns
AKT	ns	ns	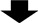
JNK	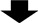	ns	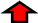
mTOR	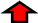	ns	ns
BDNF	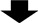	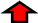	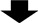

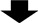
—decreased activation; 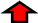
—increased activation.

**Table 2:** Summary of molecular changes in the PFC, HIPP, and STR under treatments relative to CUS.

PFC	Ket/nBNI	KET	nBNI
ERK	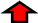	ns	ns
AKT	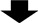	ns	ns
JNK	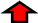	ns	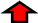
mTOR	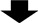	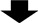	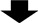
BDNF	ns	ns	ns
**HIPP**	**Ket/nBNI**	**KET**	**nBNI**
ERK	ns	ns	ns
AKT	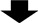	ns	ns
JNK	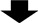	ns	ns
mTOR	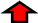 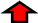	ns	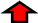 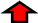
BDNF	ns	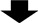	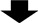
**STR**	**Ket/nBNI**	**KET**	**nBNI**
ERK	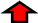	ns	ns
AKT	ns	ns	ns
JNK	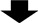	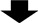	ns
mTOR	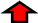 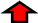	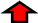 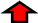	ns
BDNF	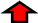 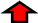	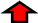	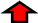 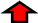

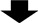
—decreased activation; 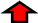
—increased activation.

**Table 3:** Types and distribution of stressors.

Day of CUS	9 a.m.	2 p.m.	6 p.m.
1			Lights on
2	Cold 4 °C, 1 h	Lights off/cage tilt, 3 h	Food deprivation
3	Cage tilt 45°, 3 h	Cold/shaking, 1 h	Stroboscope
4	Rat odor, 3 h	Swim 18°, 10 min	Wet bedding
5	Restraint, 1 h	Cold, 1 h	Cage tilt
6	Lights off, 3 h	Cold/shaking, 1 h	Lights on
7	Cold, 1 h	Rat odor, 3 h	Stroboscope
8	Restraint, 1 h	Lights off/cage tilt, 3 h	Wet bedding
9	Swim 18°, 10 min	Rat odor, 3 h	Food deprivation
10	Restraint, 1 h	Cold, 1 h	Stroboscope
11	New partner, 3 h	Restraint, 1 h	Lights on/no bedding
12	Swim, 10 min	Cold/shaking, 1 h	Wet bedding

Abbreviations—CUS: chronic unpredictable stress.

**Table 4:** Dilutions and catalog numbers of primary antibodies used for Western blot analysis.

Protein	Dilution for WB	Company	Cat. No.
p-mTOR (Ser 2448)	1:500	Santa Cruz Biotechnology, Dallas, TX, USA	sc-101738
mTOR	1:500	Santa Cruz Biotechnology	sc-517464
p-Erk1/2 (Thr202/Tyr 204)	1:1000	Cell Signaling, Danvers, MA, USA	#9101
Erk1/2	1:1000	Cell Signaling	#9102
p-Akt (Ser 473)	1:1000	Santa Cruz Biotechnology	sc-7985-R
Akt	1:1000	Cell Signaling	#9272S
p-JNK (Thr 183/Tyr 185)	1:500	Santa Cruz Biotechnology	sc-6254
JNK	1:2000	Santa Cruz Biotechnology	sc-571
BDNF	1:2000	Abcam, Cambridge, UK	ab108319

Abbreviations—p-mTOR: phosphorylated mammalian target of rapamycin; mTOR: mammalian target of rapamycin; p-Erk1/2: phosphorylated extracellular signal-regulated kinase 1/2; p-Akt: phosphorylated protein kinase B, PKB; Akt: protein kinase B, PKB; Erk1/2: extracellular signal-regulated kinase 1/2; p-JNK: phosphorylated c-Jun N-terminal kinase; JNK: c-Jun N-terminal kinase; BDNF: brain-derived neurotrophic factor.

## Data Availability

The original contributions presented in this study are included in the article. Further inquiries can be directed to the corresponding author.

## Abbreviations

The following abbreviations are used in this manuscript:

AKT	Protein kinase B, PKB
AMPA	α-amino-3-hydroxy-5-methyl-4-isoxazolepropionic acid
BDNF	Brain-derived neurotrophic factor
CUS	Chronic unpredictable stress
ERK	Extracellular signal-regulated kinase
HIPP	Hippocampus
JNK	c-Jun N-terminal kinase
Ket	Ketamine
Ket/nBNI	Combination treatment with ketamine and norbinaltorphimine
KOR	κ-opioid receptor
MDD	Major depressive disorder
mTOR	Mammalian target of rapamycin
nBNI	Norbinaltorphimine
NMDA	N-methyl-D-aspartate
ns	Not significant
OFT	Open field test
PFC	Prefrontal cortex
p-AKT	Phosphorylated protein kinase B
p-ERK	Phosphorylated extracellular signal-regulated kinase
p-JNK	Phosphorylated c-Jun N-terminal kinase
p-mTOR	Phosphorylated mammalian target of rapamycin
STR	Striatum
PND	Postnatal day
SEM	Standard error of the mean
TST	Tail suspension test
